# Auto-regulation in the powerhouse

**DOI:** 10.7554/eLife.28757

**Published:** 2017-07-10

**Authors:** Helena M Viola, Livia C Hool

**Affiliations:** 1 School of Human Sciences, The University of Western Australia, Crawley, Australia; 1 School of Human Sciences, The University of Western Australia, Crawley, Australialivia.hool@uwa.edu.au; 2 Victor Chang Cardiac Research Institute, Sydney, Australia

**Keywords:** mitochondria, mitochondrial flashes, ATP homeostasis, energy metabolism, heart, Mouse, Rat

## Abstract

Mitochondrial flashes have a central role in ensuring that ATP levels remain constant in heart cells.

**Related research article** Wang X, Zhang X, Wu D, Huang Z, Hou T, Jian C, Yu P, Lu F, Zhang R, Sun T, Li J, Qi W, Wang Y, Gao F, Cheng H. 2017. Mitochondrial flashes regulate ATP homeostasis in the Heart. *eLife* **6**:e23908. doi: 10.7554/eLife.23908

Adenosine triphosphate, or ATP for short, provides the energy that is needed for countless processes in the body. It is vital that the level of ATP in cells remains constant, especially when the demand for energy increases. This is particularly true in the heart, where energy demand can increase by a factor of 5–10 during stressful situations, yet the ATP concentration remains remarkably consistent (Balaban et al., 1986; Neely et al., 1973; Matthews et al., 1981; Allue et al., 1996).

Despite decades of research, it has remained unclear how cells keep their ATP levels stable. Now, in eLife, Heping Cheng of Peking University and colleagues – with Xianhua Wang, Xing Zhang and Di Wu as joint first authors – report that a process termed mitochondrial flash or mitoflash plays a critical role in regulating ATP concentration in the heart (Wang et al., 2017).

ATP production occurs in several stages inside mitochondria, where a flow of electrons down the mitochondrial ‘electron transport chain’ creates an electro-chemical gradient across the inner membrane of mitochondria. In the first stage, calcium is transported across the inner membrane into the inner mitochondrial chamber or matrix, and used in a process known as the citric acid cycle to generate high-energy electrons. These electrons then enter the electron transport chain and travel along the inner membrane to an enzyme called ATP-synthase that produces ATP. As a by-product of this process, molecules called reactive oxygen species are formed when electrons that leak from the electron transport chain go on to react with oxygen molecules. Although high levels of reactive oxygen species lead to cell death and disease, low levels of these species are important for regulating normal cell processes.

Recent research has shown that mitochondria exhibit brief events called mitoflashes. These involve multiple concurrent changes within the mitochondria, including a burst in the production of reactive oxygen species, as well as changes in the pH of the mitochondrial matrix, the oxidative redox state and the membrane potential (Wang et al., 2008, 2016). Mitoflashes depend on an intact electron transport chain and are thought to help regulate energy metabolism.

Using cells isolated from mouse heart muscle, Wang et al. demonstrated that it is the frequency of the mitoflashes – rather than the amplitude – that regulates ATP production. When the heart cells were exposed to drivers of the citric acid cycle to mimic increased energy metabolism, the mitoflashes occurred more frequently, while ATP production remained constant. However, when antioxidants were applied, the frequency of mitoflashes decreased, which led to an increase in ATP production. These findings suggest that mitoflash activity responds to changes in energy metabolism to negatively regulate ATP production.

When electrical stimuli were applied to make the heart cells contract more quickly and increase the demand for ATP, the frequency of mitoflashes decreased, while the cellular ATP content remained constant. It appears that when a lot of energy is needed, changes in the frequency of the mitoflashes regulate ATP production in a way that supports survival. Indeed, the results revealed that when mitoflash frequency decreased, the ATP concentration or set-point increased. This suggests that mitoflash activity may act as an ATP set-point regulator that responds to changes in energy supply and demand in order to maintain ATP homeostasis in the heart (see Figure 6 in Wang et al., 2017).

Wang et al. provide the first mechanistic insight into a potential trigger that links changes in mitoflash frequency and regulation of the ATP set-point in the heart. Previous studies have identified three possible triggers of mitoflashes: calcium located in the mitochondrial matrix, reactive oxygen species and protons (Hou et al., 2013; Wang et al., 2016). Wang et al. propose that calcium is unlikely to play a significant role in the regulation of mitoflash frequency. And since electrical stimulation did not significantly change the amount of reactive oxygen species produced by the mitochondria, they focused their attention on protons as a trigger of mitoflashes.

It is known that protons can leak through the ATP-synthase and return to the mitochondrial matrix, and it has been shown that a pro-survival protein called Bcl-xL plays a role in regulating this proton leak (Alavian et al., 2011; Chen et al., 2011). Now, Wang et al. show that an increase in Bcl-xL prevents proton leaks and reduces the frequency of mitoflashes, while the ATP set-point increases. When there is a decrease in Bcl-xL protein, the opposite occurs. Based on these findings, Wang et al. propose that proton leaks may be a bi-directional trigger of mitoflashes and cellular ATP homeostasis.

Overall, Wang et al. demonstrate that mitoflash frequency negatively regulates ATP production in a compensatory, pro-survival manner, and that a high ATP demand induces a small and brief increase in calcium. These results are consistent with previous work characterizing mitoflashes (Wang et al., 2008) and the role of mitochondria in the development of diseases in the heart (Viola et al., 2007; Seenarain et al., 2010). Future studies may provide more insight into how mitoflashes regulate ATP homeostasis during the development of heart diseases.
